# SINBAD, structural, experimental and clinical characterization of STAT inhibitors and their potential applications

**DOI:** 10.1038/s41597-022-01243-3

**Published:** 2022-03-31

**Authors:** Martyna Plens-Gałąska, Tomasz Woźniak, Joanna Wesoły, Hans A. R. Bluyssen

**Affiliations:** 1 Laboratory of Human Molecular Genetics, Institute of Molecular Biology and Biotechnology, Faculty of Biology, Adam Mickiewicz University, Poznan, Poland; 2 Institute of Human Genetics, Polish Academy of Sciences, Poznan, Poland; 3 Laboratory of High Throughput Technologies, Faculty of Biology, Adam Mickiewicz University, Poznan, Poland

**Keywords:** Virtual screening, Databases, Small molecules

## Abstract

The abnormal activation of signal transducer and activator of transcription (STAT) protein family is recognized as cause or driving force behind multiple diseases progression. Therefore, searching for potential treatment strategy is pursued by multiple scientific groups. We consider that providing comprehensive, integrated and unified dataset for STAT inhibitory compounds may serve as important tool for other researchers. We developed SINBAD (STAT INhbitor Biology And Drug-ability) in response to our experience with inhibitory compound research, knowing that gathering detailed information is crucial for effective experiment design and also for finding potential solutions in case of obtaining inconclusive results. SINBAD is a curated database of STAT inhibitors which have been published and described in scientific articles providing prove of their inhibitory properties. It is a tool allowing easy analysis of experimental conditions and provides detailed information about known STAT inhibitory compounds.

## Background & Summary

Signal transducers and activators of transcription (STATs) facilitate action of cytokines and growth factors, which are the main tool of the organism to battle any kind of immune challenge like inflammation or cancer. The STAT family consists of seven proteins: STAT1, STAT2, STAT3, STAT4, STAT5A, STAT5B and STAT6. Each STAT protein is composed of 5 domains: N-terminal, ‘coiled-coil’ (CC), DNA-binding (DBD), Src Homology 2 (SH2) and C-terminal transactivation domain. Activity of these proteins depends on Janus Kinase (JAK)-mediated tyrosine phosphorylation of a conserved tyrosine residue (pTyr) flanking the highly conserved SH2 domain. pTyr-SH2 interactions are crucial for STAT dimer formation and may further result in formation of multimeric complexes with other protein families. For a number of STATs, phosphorylation of specific serine residues has also been shown to be important^1^. Together, these active STAT complexes regulate gene transcription in the nucleus by binding to specific DNA-response elements in the promoter of their target genes. In this way, STATs facilitate action of interferons, cytokines, interleukins and growth factors and are involved in fundamental cellular processes such as cell growth, proliferation and apoptosis, embryonic development, immune responses and inflammation, and response to viral infections^2^. As a consequence, abnormal activation of STAT proteins is implicated in many human diseases, including viral and bacterial infections, inflammatory diseases, autoimmune diseases, multiple types of cancer which identifies STATs as highly attractive therapeutic targets.

Different STAT inhibitory strategies have been developed, in which STAT activity may be inhibited in a direct or indirect manner. Directly, by influencing processes such as dimerization (targeting SH2 domain) or DNA binding (targeting DNA binding domain), direct inhibition of phosphorylation (targeting transactivation domain). Or indirectly through inhibition of proteins upstream of STATs, such as the Janus kinases (JAK) JAK1, JAK2, JAK3 or Tyrosine Kinase 2 (TYK2) or of multiple interferon or interleukin receptors mediating STAT activation^3^. STAT inhibitory strategies focus on preventing STAT dimerization by using small molecules identified by *in silico* 3D modelling and virtual screening of compound libraries. As such, searches for STAT-targeting compounds, especially exploring the interaction area of the SH2 domain and the phosphorylated tyrosine residue, yielded many synthetic small molecules (over 100 compounds). Among the most potent are STA-21, STATTIC, STX-0119 and OPB-31121^4–7^. Other types of inhibitors include natural products (eg. Resveratrol and its analogues - Piceatannol and LYR71, Curcumin) peptides and peptidomimetics (CJ-1383, BP-PM, PM-73), oligodeoxynucleotide decoys and antisense oligonucleotides^8–12^.

Identification of specific and effective STAT inhibitory strategies could provide a tool to increase our understanding of their functional role in different diseases. Moreover, promising results for several STAT inhibitors in recent clinical trials predicts STAT-inhibiting strategies may find their way to the clinic, and could serve as therapeutic strategies in cancer, inflammation, autoimmunity and viral infections. Since December, 2019, a disease outbreak caused by a novel coronavirus (SARS-CoV-2) was declared a global public health emergency by WHO and named coronavirus infected disease-19 (COVID-19)^13^. Dysregulated host immune responses and robust production of inflammatory cytokines and interferons, known as the “cytokine storm”, correlate with disease severity and poor prognosis during SARS-CoV2 infection^14^. Since many of these factors are potent activators of STAT signaling pathways, this identifies STATs as potential therapeutic targets in COVID-19 disease as well. Anti-IL6 antibodies, as well as JAK inhibitors (indirect STAT inhibitors)- Baricitinib, Fedratinib, and Ruxolitinib have already been selected as part of a potential treatment strategy against COVID-19 as a combined antiviral and anti-inflammatory approach^15–19^. Ruxolitinib entered phase III clinical trial (NCT04120090, NCT03533790) and Fedratinib phase II, both were used in pneumonia associated COVID-19 cases.

The relevance of STATs as therapeutic targets is emphasized by the numerous studies and publications of STAT inhibitors, involving multiple *in silico*, *in vitro*, *ex vivo*, *in vivo* methods, in different experimental settings and disease models and the inclusion of a number of these inhibitors in clinical trials. With our database we provide a comprehensive tool for detailed characterization of compounds disrupting STAT signaling in various conditions allowing better understanding of their nature and mode of action. In addition, our database can be a source of information for other groups and function as primary selection tools for potential known inhibitors for further investigation in SARS-CoV-2 research.

## Methods

### Data collection

**Fig. 1:**
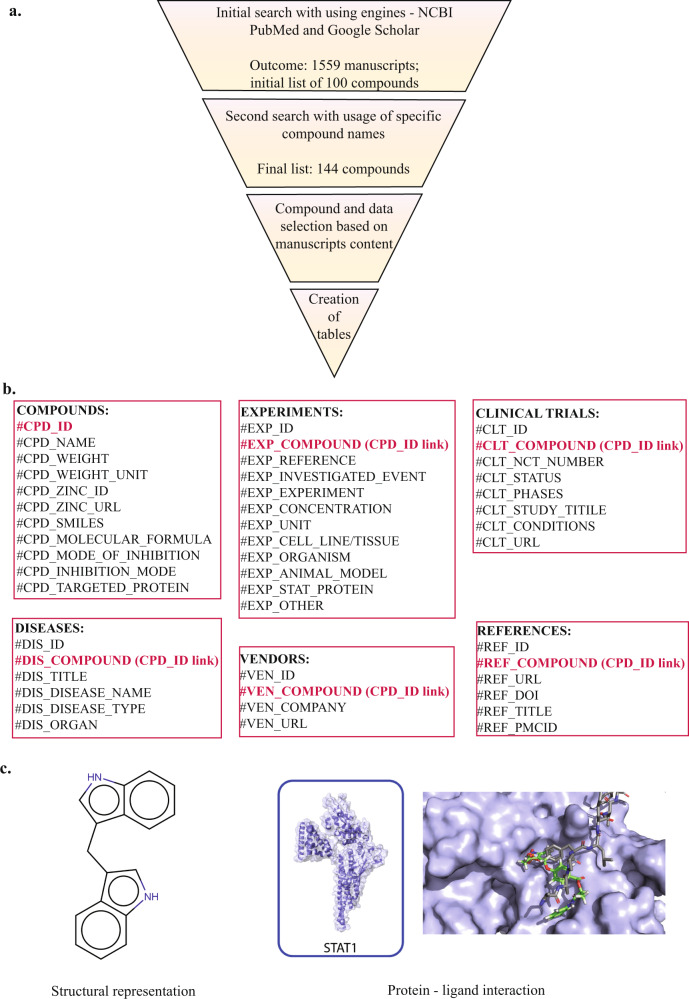
Methodology of data collection and categorization. (**a**) Presentation of workflow for data collection. Gathered data was examined and categorized allowing to establish final list of 141 inhibitory compounds. (**b**) Outline of tables used for creation of SINBAD. Gathered data was put into 6 tables which are linked with each other (marked with red) by one variable - specific number each compound was given in initially created table COMPOUNDS. (**c**) Graphical data available in SINBAD. We provide structures of compounds and for some we visualized ligand – protein interaction using STAT-SH2 3D models and Surflex -Dock 2.6.

We created the SINBAD Database following guidelines described by the FAIR data principles^20^. An initial inhibitor list was created based on a collection of review articles selected in the search described below. This was followed by manual selection of suitable research manuscripts which further were divided into small groups based on the described inhibitors. Using scientific search engines - National Center for Biotechnology Information (NCBI) - PubMed and Google Scholar, firstly we focused on gathering names of known STAT inhibitory compounds. For this purpose, we initially used available Review articles which summarized STAT inhibitory strategies with a description of exemplary inhibitors, many of which focused on cancer research. This was followed by using more advanced search and phrase-base options: “(“stat1 inhibitor”) OR (“stat2 inhibitor”) OR (“stat3 inhibitor”) OR (“stat4 inhibitor”) OR (“stat5 inhibitor”) OR (“stat5a inhibitor”) OR (“stat6 inhibitor”) OR (“stat1 inhibition”) OR (“stat2 inhibition”) OR (“stat3 inhibition”) OR (“stat4 inhibition”) OR (“stat5 inhibition”) OR (“stat5a inhibition”) OR (“stat6 inhibition”). In this way, from PubMed we extracted 1559 potential literature sources for our database. Additionally, we checked separately every compound by its name in both NCBI PubMed and Google Scholar. This approach allowed us to thoroughly screen available literature, followed by initial manual screening of each publication and further selection and proper grouping (Fig. 1a). In the SINBAD database, we decided to include inhibitors: small compounds, antibodies, peptides, peptidomimetics, oligonucleotides which interact directly with STAT proteins but also those which may influence STATs indirectly by interacting with other proteins for instance targeting JAK kinases or multiple interferon or interleukin receptors. Where possible we provided information about which protein or which protein domain was targeted or was proposed to interact with the inhibitor. In case where we could not find detailed data, we used more general terms eg. “JAK-STAT inhibition”, “JAK inhibitor”, “STAT downregulation”, or did not provide any information. In case of a proven interaction of the inhibitor with the STAT-SH2 domain, we provided additional docking visualization (Fig. 1c) by using the Surflex-Dock 2.6 program in combination with STAT 3D models which were previously published by or group^21^. What is more, we decided to include information from publications which described only STAT inhibition and to omit publications which predominantly focused on other proteins, transcription factors or pathways. This approach allowed us to build an initial inhibitor list consisting of approximately 100 STAT inhibitors and then by further investigation we composed a final list with 144 positions described in over 200 publications (Fig. 1a).

## Data Records

### Database design

The datasets generated and analyzed in this study are available at http://sinbad.amu.edu.pl as well as through public repository 10.6084/m9.figshare.14975136.v1^22^. In SINBAD, we collected crucial experimental data describing detailed characteristics of each individual inhibitor. Datasets can be obtained either via SINBAD webpage or via Figshare Repository. The Repository has folder structure. The code is available via the paths ‘stat_project/stat_database’ and ‘stat_project/apps’, both containing python files creating the project additionally through the path ‘stat_project/templates’ we provide access to.html files. All of the additional files and external libraries used for the graph management, table visualization are located in ‘static’ folder. The data itself is localized in ‘stat_project\apps\stats\management\data’ and ‘stat_project/media’ containing Excel file with tables on which the database is built, and all used structural representations respectively. Variables gathered in tables are preceded by a prefix determining the origin of the variable. Data is summarized in 6 Excel tables: COMPOUNDS (CPD), EXPERIMENTS (EXP), VENDORS (VEN), REFERENCES (REF), CLINICAL_TRIALS (CLT), DISEASES (DIS). Each compound was given its own unique ID number (CPD_ID) which allowed us to easily form interactions between the tables. Table COMPOUNDS contains 175 inhibitors with their basic features such as ID number, name (CPD_NAME), weight (CPD_WEIGHT), weight unit (CPD_WEIGHT_UNIT), ZINC ID (CPS_ZINC_ID), SMILES code (CPD_SMILES), CAS number (CPD_CAS), molecular formula (CPD_MOLECULAR_FORMULA), mode of inhibition, (CPD_MODE_OF_INHIBITION)) inhibited target (CPD_TARGETED_PROTEIN). More importantly, the core information of the database is gathered in the table EXPERIMENTS which consist of over 20,500 records providing data about the experimental approach used to characterize each inhibitor. Within the table there are variables such as record number (EXP_ID), compound number at the original list (EXP_COMPOUND originating form CPD_ID) reference number given in the table REFERENCES (EXP_REFERENCE), name of the investigated event/process (EXP_INVESTIGATED_EVENT), type of experiment (EXP_EXPERIMENT), concentrations of tested compounds (EXP_CONCENTRATION), unit of concentration (EXP_CONCENTRATIONS), cell line or tissue types used in experiment (EXP_CELL_LINE/TISSUE), organism that tissues or cell lines originated from (EXP_ORGANISM), animal model used in *in vivo* testing (EXP_ANIMAL_MODEL), investigated STAT protein (EXP_STAT protein). To make this table searchable and functional we decided to use two variables: INVESTIGATED_EVENT and EXPERIMENT (Fig. 1b) instead of one. This approach allowed us to describe in more detail the experiment itself and to distinguish between different conditions/parameters, for example apoptosis may be monitored by various types of experiments such as Western blot, Flow cytometry, MTT or MTS assays. Western blot, on the other hand, is a widely used technique that illustrates protein activity in various cells and tissues. Together these variables create an easy and user-friendly way to delve into the details of each publication and compare presented results in a comprehensive way. Two smaller tables REFERENCES and VENDORS provide links to NCBI PubMed, title of publication, unique DOI number and PMC ID (REF_ID, REF_COMPOUND, REF_URL, REF_DOI, REF_TITLE) and links to potential vendor web pages (VEN_ID, VEN_COMPOUND, VEN_COMPANY). To provide a more complete picture, in the CLINICAL_TRIALS table, for some compounds we present data regarding clinical trials that have been performed and documented (over 16500 trials). Within this table we gathered basic information such as unique number of conducted trial (CLT_NCT_NUMBER) followed by current trial status and phase (CLT_STATUS, CLT_PHASE). Further we provide data regarding title of the conducted study and investigated diseases (CLT_STUDY_TITLE, CLT_CONDITIONS) and a link to the full report from the conducted study (CLT_URL). Of course, only some inhibitors were pursued into clinical trials, therefore clinical trial data is available only for a few of presented inhibitors. Furthermore, in DISEASE table, we compiled information from the same sources as used for the other tables about potential disease treatment strategies in which the inhibitor of interest was used (DIS_DISEASE_NAME, DIS_DISEASE_TYPE).

## Technical Validation

For the database creation we used Django web framework, Docker for efficient deployment, Nginx as web server, Elasticsearch as a search engine and finally MariaDB as SQL database.

## Usage Notes

**Fig. 2:**
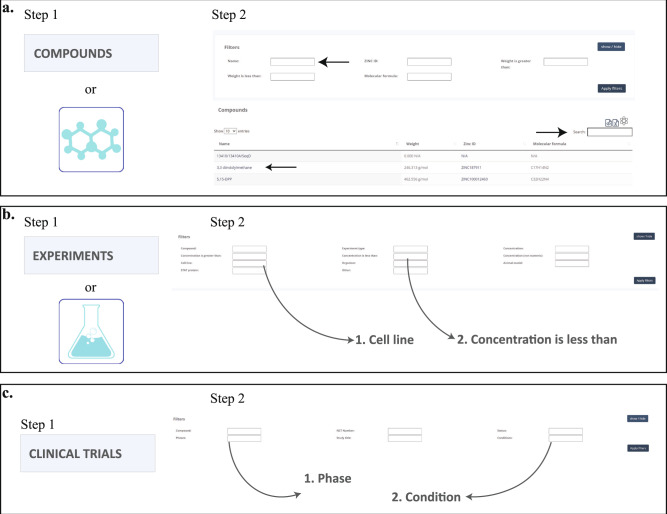
Exemplary usage of SINBAD. (**a**) Question: I am looking for all data gathered for STAT inhibitor. (**b**) Question: I am interested in compounds which were tested in Hela cell line with concentration lower than 50 µl. (**c**) Question: I am looking for Clinical Trial data for all compounds that entered Phase I clinical trials investigating breast cancer.

The SINBAD database provides multiple options of filtering or searching depending on the individual users’ preference – it can be used as a dataset downloaded on a personal computer and managed with R, Excel or used online. With SINBAD the user can address multiple questions regarding STAT inhibition and conditions in which it was tested (exemplary webpage layout described in Supplementary Data and shown in Supplementary Fig. 1). It will allow to establish better conditions for future experiments and prevent repeating already existing data. In Fig. 2 we show exemplary questions which can be answered with SINBAD. If the User wants to retrieve all available data about a specific compound he/she has to choose at the homepage either COMPOUND in the left Menu panel or the molecule symbol on that page (Fig. 2a, Step 1). This will transfer the User to the table summarizing inhibitory compounds gathered in the database. Step 2 – using either filtering options, type the name of the compound in the search window (marked with arrow) or choose compound from the list below (Fig. 2a, Step2). On the other hand, if the User wants to investigate which compounds were tested in HeLa cell line at 50μM concentration, he/she has to choose at the homepage either EXPERIMENTS in the left Menu panel or the Dish symbol on this page (Fig. 2b, Step 1). This will transfer the User to the table summarizing experimental data gathered in the database. Step 2 -using filtering options, type the cell line name - marked as 1, and concentration in proper filter window - marked as 3 (Fig. 2b, Step 2). Finally, if the User wants to search for data for a compound that entered Phase I clinical trials for breast cancer, he/she has to choose CLINICAL TRIALS (Fig. 2c, Step 1). This will transfer the User to the table summarizing clinical trial data gathered for inhibitory compounds. Step 2 -using filtering options type number of phase of interest in window marked as 1. and specify condition using filter marked as 2 (Fig. 2c, Step 2).

The SINBAD database is constantly being updated by the administrator of our group. What is more, it is possible for external users to upload their own published results, for which we provided a simple procedure. The User can request to add their own published data through a special contact form through which the User will receive access to a dedicated uploading panel. However, unpublished data will first have to be verified and approved by the administrator. One limitation of our dataset is that it does not include publications focusing on non-STAT target proteins. We are aware that there are multiple publications covering inhibitory properties of presented inhibitors that target pathways other than JAK-STAT. We are planning to expand SINBAD with additional experimental data gathered form publications focusing on non-STAT targets, including transcription factors such as IRFs, NF-κB and others.

## Supplementary information

Supplementary Data Figure 1

Supplementary Data

## Data Availability

All generated code and data are hosted within Figshare repository 10.6084/m9.figshare.14975136.v1^22^.
