# Supplementary material for: SINBAD, structural, experimental and clinical characterization of STAT inhibitors and their potential applications
Source: Sci Data. 2022 Mar 31;9:139. doi: 10.1038/s41597-022-01243-3 (PMC8971479; doi:10.1038/s41597-022-01243-3)
Supplement: Supplementary file 1 — Supplementary Data Figure 1 [file 41597_2022_1243_MOESM1_ESM.pdf]

3,3 diindolylmethane

a.

Name	Value
Weight	246.313 g/mol
Zinc ID	ZINC187911
Smiles	c1ccc2c(Cc3c[nH]c4ccccc34)c[nH]c2c1
Molecular formula	C17H14N2
Mode of inhibition	N/A

b.

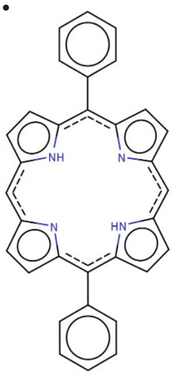

Clinical Trials

c.

Show 10 entries

Search:

Study title	Status	Conditions	Link
3,3'-Diindolylmethane in Patients With Systemic Lupus Erythematosus	Terminated	SLE	NCT02483624
A Nutritional Intervention to Decrease Breast Density Among Female BRCA (BReast CAncer Susceptibility Gene) Carriers	Unknown status	BRCA1 Gene Mutation|BRCA2 Gene Mutation	NCT02197000
Diindolylmethane in Healthy Volunteers	Completed	Healthy, no Evidence of Disease	NCT00392652

Visualization

d.

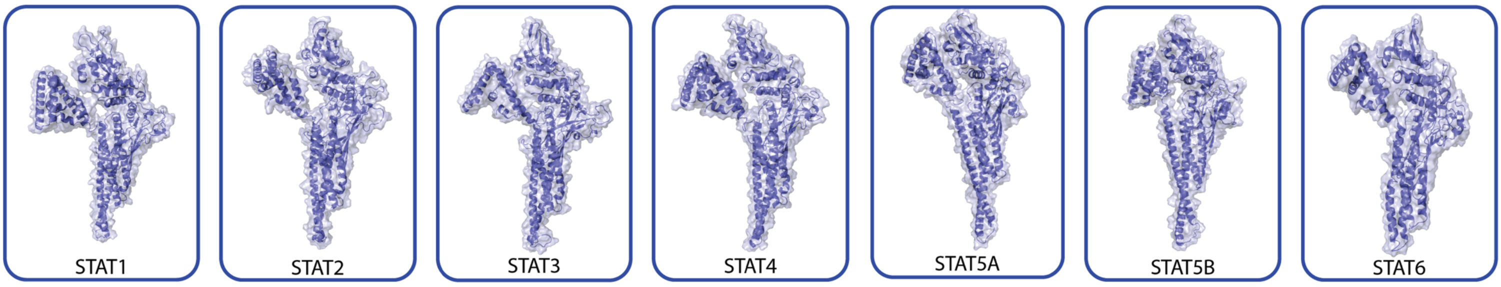

References

e.

Show 10 entries

Search:

Publication	DOI	Pubmed	PMCID
**3,3'Diindolylmethane suppresses vascular smooth muscle cell phenotypic modulation and inhibits neointima formation after carotid injury.** *PloS one* Hongjing Guan, Lihua Zhu, Mingyue Fu, Da Yang, Song Tian, Yuanyuan Guo, Changping Cui, Lang Wang, Hong Jiang, 7, 4, None, 2012-04-10, 10.1371/journal.pone.0034957	10.1371/journal.pone.0034957	22506059	PMC3323601
**Diindolylmethane suppresses ovarian cancer growth and potentiates the effect of cisplatin in tumor mouse model by targeting signal transducer and activator of transcription 3 (STAT3).** *BMC medicine* Prabodh K Kandala, Sanjay K Srivastava, 10, None, 2012-01-26, 2012-01-26, 10.1186/1741-7015-10-9.	10.1186/1741-7015-10-9.	22280969	PMC3298725
Inhibition of STAT Signalling in Bladder Cancer by Diindolylmethane - Relevance to Cell Adhesion, Migration and Proliferation	N/A	N/A	N/A
Regulation of Janus-activated kinase-2 (JAK2) by diindolylmethane in ovarian cancer in vitro and in vivo	10.5582/ddt.2012.v6.2.94	https://www.ncbi.nlm.nih.gov/pubmed/22622019	N/A

Showing 1 to 4 of 4 entries

Previous 1 Next

Vendors

f.

Company	Lnk to product
Caymanchemical	https://www.caymanchem.com/product/16090/5%2C15-dpp
MERCK (Sigma Aldrich)	https://www.sigmaaldrich.com/catalog/product/mm/573109?lang=pl&region=PL
Sanata Cruz Biotechnology	https://www.scbt.com/p/stat3-inhibitor-viii-5-15-dpp-22112-89-6
Abcam	https://www.abcam.com/515-dpp-stat3-inhibitor-viii-stat3-inhibitor-ab146023.html
CliniSciences	https://www.clinisciences.com/autres-produits-186/stat3-inhibitor-viii-5-15-dpp-22112-1275971.html

Filter data

g.

Compound	3,3 diindolylmethane
Experiment types	Aortic ring assay , BrdU cell proliferation assay, Caspase 3/7 activity, Cell Migration Assay , Collagen adhesion assay , ELISA, EZ-TFA transcription factor colorimetric assay, Flow cytometry , Flow cytometry- annexin V binding and propidium iodide staining, Immunofluorescence, Immunohistochemical staining, Immunohistochemistry, Luciferase activity, Mice weight, SRB cell survival assay, TUNEL, Transwell Boyden Chamber; Hema Gurr rapid stain (BDH), Trypan blue dye exclusion assay, Tumor volume, WST-1, Western Blot, Wound healing assay, annexin V binding and propidium iodide staining
Concentrations	1.00, 10.00, 100.00, 120.00, 150.00, 20.00, 25.00, 3.00, 40.00, 5.00, 50.00, 60.00, 75.00, 80.00
Cell lines	A2780, HUVECS, J82, NOSE, OVCAR-3, OVCAR-429, OVCAR-433, RT112, RT4, SKOV-3, SKOV-3 (xenograft), SKOV-3 (xenografts), TOV-21G, VSMC, carotid artery tissue, rat thoracic aorta
Organisms	Homo sapiens, Mus musculus, Rattus norvegicus
Animal models	Crl:NU(NCr)-Foxn1nu, athymic nude mice, c57BL/6
STAT proteins	PARP, STAT1, STAT1 (cytoplasm), STAT1 (nucleus), STAT3, STAT3 (cytoplasm), STAT3 (nucleus), STAT5A, STAT5B, p-STAT1 (Y701), p-STAT1 (cytoplasm), p-STAT1 (nucleus), p-STAT3, p-STAT3 (S727), p-STAT3 (Y705), p-STAT3 (Y705) (nucleus), p-STAT3 (cytoplasm), p-STAT3 (nucleus), p-STAT3(Y705)
Other	Actin, Akt473, CD31, CD45, CDK4, CDK6, Cdc25C, Cdk1/Cdc2, Cdk6, Cleaved Caspase 3, Cleaved Caspase-3, Cleaved PARP, Cyclin B1, Cyclin D1, Desmin, ERK, GAPDH, GSK3β, HIF-1α, IL-6, Lamin B (nucleus), Mcl-1, PDGFR, Plk1, SM α-actin, SM22, Survivin, VEGF, actin, lamin (cytoplasm), lamin (nucleus), p-Akt473, p-ERK, p-GSK3β, p-JAK2 (Y1007), p-PDGFR, p-cofilin, p27Kip1

Experiments

h.

Show 10 entries

Search:

Compound	Experiment type	Concentration	Cell line	Organism	Animal model	Stat protein	Details
3,3 diindolylmethane	Western Blot	25.00 μM	SKOV-3	Homo sapiens			
3,3 diindolylmethane	Western Blot	50.00 μM	SKOV-3	Homo sapiens			
3,3 diindolylmethane	Western Blot	75.00 μM	SKOV-3	Homo sapiens			
3,3 diindolylmethane	Western Blot	25.00 μM	NOSE	Homo sapiens			

Supplementary Figure 1. Exemplary page presenting data about STAT inhibitory compound in SINBAD.

A. Summary table with basic data regarding name, formula, molecular weight and way compound interacts with STAT proteins. **B.** Presentation of molecules' structure. **C.** Searchable table with data regarding performed clinical trials (if available). It provides name of conducted study with its unique number, information about status and conditions of particular trial. **D.** For some compounds we provide visualization of their interaction with STAT-SH2 domain. Models published in PlosOne by Szelag et al. **E.** References with links to Pubmed and DOI numbers. **F.** Section providing quick links to potential vendor webpages. **G**. Table summarizing all conducted experiments and all conditions that were tested including cell lines, compound concentrations, animal models. STAT proteins and other possible targets. Each phrase is a functional link to more detailed information about particular conditions. **H.** Searchable table containing detailed information about all experiments conducted with usage of a particular compound.
