# Supplementary material for: SINBAD, structural, experimental and clinical characterization of STAT inhibitors and their potential applications
Source: Sci Data. 2022 Mar 31;9:139. doi: 10.1038/s41597-022-01243-3 (PMC8971479; doi:10.1038/s41597-022-01243-3)
Supplement: Supplementary file 2 — Supplementary Data [file 41597_2022_1243_MOESM2_ESM.pdf]

SUPPLEMENTARY DATA.

Webpage design. SINBAD is freely available at http://sinbad.amu.edu.pl. It was created to allow researchers to search through all available data from the field of STAT inhibitor research and to perform effective data mining and analysis in a quick and user-friendly manner. For each inhibitor we created a separate page (Supplementary Figure 1) containing crucial characteristics including its chemical features, structure and other literature-based data. At the top of each page, we provided basic information in the form of tables, the first one contains basic chemical features such as molecular weight and structural information in the form of formula, SMILES code and visualization (Supplementary Figure 1A, B). This is followed by the table containing clinical trial data if these records were available. It consists of current trial status, its title, and also includes conditions which were the main focus of the trial. For each trial we provided its unique number with cross-reference to the clinicaltial.gov webpage (Supplementary Figure 1C). In case an inhibitor was identified as a direct SH2 domain inhibitor we also provide a visualization option of the STAT-inhibitor interaction (Supplementary Figure 1D). All gathered data is supported by the references and information about vendors where possible (Supplementary Figure 1E, F). Tables presenting information about experimental data and clinical trials are suitable for quick and easy filtering to allow more adequate data mining and extraction (Supplementary Figure 1G). To facilitate a search for specific entries, multiple filters can be applied. With the available options the user is not limited to the use of names of experiments but also may search for more general terms and find multiple types of experiments conducted to investigate one type of event (Supplementary Figure 1H). For example, if the user is interested in "protein-DNA interaction" and will use that phrase in the search option, it will provide different types of experiments and their conditions such as Electromotility Shift Assay (EMSA), chromatin immunoprecipitation etc. For another example, there is a possibility to search for a specific interaction between a STAT domain and the inhibitor, or even search for non-STAT protein inhibition involved in the JAK-STAT pathway. Basic chemical data including molecular weight, structure, formula, SMILES code is available for each compound.
